# FROG: a new people detection dataset for knee-high 2D range finders

**DOI:** 10.3389/frobt.2025.1671673

**Published:** 2025-10-20

**Authors:** Fernando Amodeo, Noé Pérez-Higueras, Luis Merino, Fernando Caballero

**Affiliations:** Service Robotics Lab, Universidad Pablo de Olavide, Seville, Spain

**Keywords:** human-aware robotics, 2D LIDAR, people detection, dataset, ROS, benchmark, deep learning

## Abstract

Mobile robots require knowledge of the environment, especially of humans located in its vicinity. While the most common approaches for detecting humans involve computer vision, an often overlooked hardware feature of robots for people detection are their 2D range finders. These were originally intended for obstacle avoidance and mapping/SLAM tasks. In most robots, they are conveniently located at a height approximately between the ankle and the knee, so they can be used for detecting people too, and with a larger field of view and depth resolution compared to cameras. In this paper, we present a new dataset for people detection using knee-high 2D range finders called FROG. This dataset has greater laser resolution, scanning frequency, and more complete annotation data compared to existing datasets such as DROW (Beyer et al., 2018). Particularly, the FROG dataset contains annotations for 100% of its laser scans (unlike DROW which only annotates 5%), 17x more annotated scans, 100x more people annotations, and over twice the distance traveled by the robot. We propose a benchmark based on the FROG dataset, and analyze a collection of state-of-the-art people detectors based on 2D range finder data. We also propose and evaluate a new end-to-end deep learning approach for people detection. Our solution works with the raw sensor data directly (not needing hand-crafted input data features), thus avoiding CPU preprocessing and releasing the developer of understanding specific domain heuristics. Experimental results show how the proposed people detector attains results comparable to the state of the art, while an optimized implementation for ROS can operate at more than 500 Hz.

## 1 Introduction

Nowadays, mobile robots are becoming part of our daily lives. Robots must be capable of sharing the space with humans in their operational environments. Therefore, human social conventions must be taken into account when navigating within the scenario in order to improve people’s comfort. The first step to achieve this is human perception. Robots must be able to detect people in their surroundings, distinguishing them from other static and dynamic obstacles.

In the last few years, image-based algorithms for detection and tracking of people have evolved significantly. Moreover, these algorithms can also work in 3D space by using cameras with depth perception. However, the use of these cameras for human detection in the robot navigation task still presents some drawbacks. The field of view of most cameras is very limited, and so is the depth perception range. Robots usually work around these limitations by making use of several cameras, which thus increases the complexity of the system and the computation requirements.

On the other hand, most commercial and non-commercial ground robots include 2D LiDAR range finders. This includes industrial robots used in warehouses such as autonomous mobile robots (AMRs) and automated guided vehicles (AGVs), which must perform people detection if their specification calls for sharing the operating environment with human workers. In any case, another major driving motivation for 2D LiDAR usage is reducing the total cost of the robot, while still providing a platform that can reliably perform obstacle detection and robot localization. The cost of 2D LiDAR sensors has gone down in recent years, and economical models for hobbyist/educational use can even be found in mainstream marketplaces. In addition, LiDARs provide accurate range measurements closely achieving full 360° coverage as well as long range detection, making them a good choice for the aforementioned use cases. Furthermore, in most cases, these sensors are installed on robots at a plane parallel to the ground that is approximately knee height.

A number of robotics researchers have worked on using 2D range finders to detect people in the proximity of a robot. The first approaches used hand-crafted features and classical algorithms (Arras et al., 2007; Pantofaru, 2010), while later approaches employed deep learning techniques (Beyer et al., 2017; 2018; Guerrero-Higueras et al., 2019; Jia et al., 2020; 2021). However, most publicly available datasets for people detection in robotics involve other kinds of sensors, or require relabeling. Very few datasets specifically geared for 2D range finders exist, the most notable of which is the DROW dataset (Beyer et al., 2017; 2018).

This work aims to fill this gap by releasing a completely new 2D range finder dataset specifically focused on person detection. The laser scans were recorded as part of the Fun Robot Outdoor Guide (FROG) project (Evers et al., 2014). The scenario consists of a tour of the Royal Alcázar of Seville, an iconic Mudéjar palace receiving over 1.5 million visitors a year. Our dataset contains a large number of laser scans, and unlike Beyer et al. (2017) every single one is annotated. This is possible using our semi-automatic annotation tool, which considerably reduces the workload required to annotate such an extensive dataset.

Overall, we present the following contributions:

• A fully annotated 2D range finder person detection dataset including a variety of indoors and outdoors scenes, crowded scenes, and challenging features (such as pillars, bushes, slopes, etc.). This dataset contains a total of over 400k LiDAR scans, all of which are annotated (compared to DROW, which only annotates 5%), a total of over 1 million people annotations, around 3 h of recorded time, and a total travel distance of over 10 km.
• A deep learning based people detection model that learns people-distinguishing features directly from the range data vector without requiring a preprocessing step, and produces person location proposals using techniques analogous to those of image-based object detectors. Our optimized ROS-based implementation also achieves inference times of less than 2 ms, which is considerably faster than the best currently available solutions.
• A benchmarking codebase and methodology complementing and supporting our dataset, which allows researchers to evaluate their own 2D range finder person detectors under standardized metrics and metric computation code.

## 2 Related work

In this section we survey existing datasets related to people detection and range finders, with attention to their composition and attributes. We also survey existing works that aim to detect people using these sensors. Our findings, detailed below, show that most datasets are either geared towards autonomous driving tasks (with limited genericity and relevance to people detection), or involve other kinds of sensors. People detectors also tend to be based on classical algorithms or make use of hand-crafted input processing. We focused on studying detectors involving the use of deep learning, even if they also contain non-deep processes.

### 2.1 Annotated 2D LiDAR datasets

There are many datasets with people annotations in the form of bounding boxes or segmentation masks, geared towards plain 2D images or sensors such as RGBD cameras or 3D LiDAR. However, there is a scarcity of people detection datasets geared towards 2D LiDAR sensors, containing annotated 2D range data.

With the rise of autonomous (self-driving) cars, several multimodal datasets have been recorded and released, such as nuScenes (Caesar et al., 2020), KITTI (Geiger et al., 2013), and PedX (Kim et al., 2019). These datasets focus on traffic scenes, where most objects on the roads are vehicles, and pedestrians are sparsely distributed.

Focused on common pedestrian situations in indoor and outdoor environments, we found datasets with pedestrians annotated in images and 3D LiDAR point clouds like JRDB (Martín-Martín et al., 2023), SCAND (Karnan et al., 2022), STCrowd (Cong et al., 2022) or WILDTRACK (Chavdarova et al., 2018) (the latter only based on static camera images in outdoor scenarios). There also exist 2D LiDAR datasets for general purpose segmentation such as Semantic2D (Xie and Dames, 2024), however they contain many more classes besides “person”, and thus they are not well suited for training people detectors.

Other datasets provide annotated trajectories of human pedestrians performed in a unique controlled laboratory environment like the THÖR dataset (Rudenko et al., 2020) or the Magni Human Motion dataset (Schreiter et al., 2022). These datasets are dedicated to learning social navigation (as opposed to simply people detection), and only the latter provides data from a robot’s mounted 2D LiDAR sensor.

In this work, we focus on datasets with annotated pedestrians in 2D laser scans, and people detectors based on the same sensory input. The DROW dataset was introduced in Beyer et al. (2017) for the detection of wheelchairs (wc) and walking aids (wa) in laser scan data. The authors recorded 113 sequences at an elderly care facility. In a follow-up work (Beyer et al., 2018), the authors added person (wp) annotations. We found the following drawbacks in the dataset:

• During the annotation process, the scans were batched in groups of 100, and only 1 out of 4 batches was provided to human volunteers. Moreover, within each batch, only 1 out of 5 scans was annotated. This combination results in just 1 out of 20 scans (5% of the total) carrying annotations, with the remaining 95% being left completely unannotated. Even though the authors justified this decision in reducing the workload of the annotators, as well as reserving the unannotated scans within each batch for temporal approaches; it still means a large majority of the data is unusable for direct supervised learning, reducing the variability of input samples and prompting the use of data augmentation. In addition, temporal approaches such as DR-SPAAM (Jia et al., 2020) do not necessarily follow the prescribed temporal window stride hyperparameter, instead experimenting with different strides (such as T=10 ).
• Despite the authors’ efforts in adding people annotations, the dataset is still mainly focused on detecting mobility aids, meaning the amount and quality of person annotations is inadequate for other use cases, compromising the genericity of the dataset.
• As pointed out by Jia et al. (2020), the validation set is considerably more challenging than the training or test sets because it contains more people annotations at farther distances (meaning sparser points). This causes problems during hyperparameter search, and can also lead researchers to make mistakes when trying to assess any possible overfitting.

Another recently available 2D LiDAR dataset for people detection is Sixth Sense (Arreghini et al., 2025). This dataset leverages an additional Azure Kinect sensor to produce unsupervised people detections. However, the dataset is very short (around 55k scans at 10 Hz), and it is recorded fully indoors within a university campus. Moreover, the person detection data is not directly in the form of annotations, instead being presented as the output of 360 ° human distance/presence detectors; thus requiring further processing to separate each person instance and generate person annotations usable with existing detectors.

### 2.2 People detectors

There are plenty of different detectors and trackers of people based on images and depth perception, as commented previously. These include face detectors, full-body detectors, or even skeleton detectors. However, the field of people detection in 2D range data has not been thoroughly explored and researched. We consider this to be related to the complexity of the problem, given the scarcity of reliable information that can be extracted from the range data in order to detect people.

Arras et al. developed a segment-based classifier that detected people’s legs using hand-crafted features extracted from each segment (Arras et al., 2007). Later, an implementation of Arras’s leg detector classifier was released for its use with the ROS middleware (Pantofaru, 2010).

Particularly, we are interested in more novel approaches based on Deep Learning. The PeTra (People Tracking) detector (Guerrero-Higueras et al., 2019) replaced the shallow learning based algorithm in Arras’s leg detector with a deep 2D fully convolutional segmentation network (using a projected 2D occupancy map of the range data as input), while still maintaining a classical post-processing step for extracting locations of individual legs from the segmentation output, as well as combining legs into person detections. The authors also propose using a Kalman filter to produce smoother person tracking over time.

The DROW (Distance RObust Wheelchair/Walker) detector (Beyer et al., 2017; 2018) proposes creating many small fixed-size windows centered around every point of the scan called “cutouts”, which are normalized to contain the same fixed number of range values. This eliminates the spatial density variability problem caused by laser points at different distances. Then, a 1D convolutional network is used to extract features from each cutout, and decide both whether a person is nearby, as well as regress a spatial offset to said person. The regressed spatial offsets are taken as votes, and used to refine the final detected location of the person. The network is trained with a dataset of range data created and labeled by the same authors (DROW dataset). The authors then followed up with an improved version of their detector that fuses temporal information (Beyer et al., 2018), and spatially aligning cutouts with those of recent past scans with the help of odometry data from the robot.

A newer people detector work (Jia et al., 2020) proposes Distance Robust Spatial-Attention and Auto-regressive Model (DR-SPAAM). Similar to DROW, it continues to use cutouts of the laser scan data, but also using a forward looking paradigm to aggregate temporal information. Instead of computing spatially aligned cutouts on the past scans, it uses a similarity-based spatial attention module, which allows the CNN to learn to associate misaligned features from a spatial neighborhood. The same authors also present a self-supervised approach of the DR-SPAAM detector (Jia et al., 2021) in which a calibrated camera with a conventional image-based object detector model is initially used to detect the people in the scene, and subsequently used to generate “pseudo-labels” in the range data for self-supervised learning.

Finally, more recent works include Li2Former (Yang et al., 2024), which replaces the traditional CNN used by the cutout-based approach with a Transformer-based architecture; however at the expense of increased model and training complexity, and heavier runtime processing leading to decreased speed. Moreover, to the best of our knowledge there are no publicly available implementations of this detector ready for use by robotics researchers.

A general trend in the surveyed detectors is the combination of a deep learning network with non-deep pre-processing and post-processing steps. In particular, the cutout-based approach increases the dimensionality of the input by virtue of generating as many cutouts as there are points in each range data vector. Likewise, the temporal approach involves aggregating data from several scans at once, possibly with alignment steps (both deep and non-deep). This in particular is used to justify the reduced annotation coverage of the DROW dataset, which significantly harms the development of detectors based on more direct approaches. These factors all result in increased memory and computational overhead at both training and inference time–according to Jia et al. (2020), their baseline implementation of the DROW detector (without temporal aggregation) needs 97.4 ms per scan (10.4 FPS) on an edge device suitable for robotics (Jetson AGX), while a special “faster” implementation of DR-SPAAM called “DR-SPAAM*” needs 44.3 ms per scan (22.6 FPS). One of the motivations of this work is encouraging further research into removing the need for these non-deep steps, and specifically in Section 4 we will propose an initial approach into a fully deep person detector based on 2D range data.

## 3 FROG dataset

The FROG dataset is a large dataset of people detection in 2D LiDAR data covering a populated public space, the Royal Alcázar of Seville. The Alcázar is a UNESCO World Heritage Site famous for its Mudéjar Hispano-Muslim architecture and its verdant gardens and courtyards, which receives over 1.5 million visitors a year–one of the most visited monuments in Spain. As a result, the dataset presents a rich variety of highly populated areas and scenarios, both indoors and outdoors.

The FROG dataset for 2D laser people detection is available for download from our website^1^. The source of the data is our previous dataset (Ramón-Vigo et al., 2014), which contains a larger collection of raw sensor data appropriate for localization and human-robot interaction purposes. This data was also previously made publicly available^2^.

**FIGURE 1:**
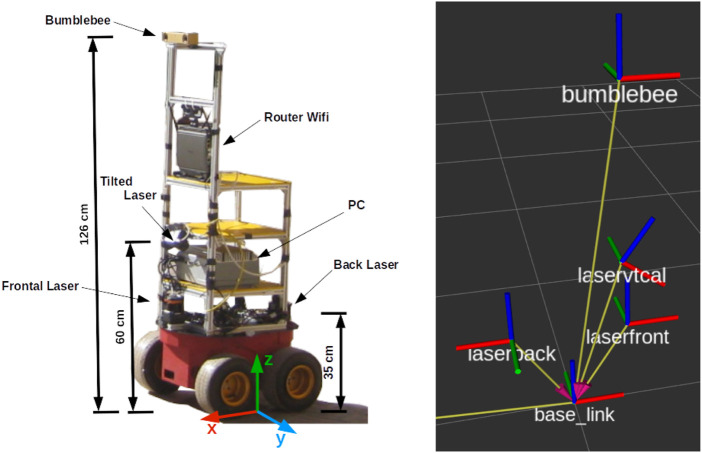
Left: image of the robot platform used for recording. Right: reference frames of the robot. The front mounted 2D LiDAR sensor (laserfront) is placed at X = 0.22 m and Z = 0.33 m with respect to the base of the robot (base_link).

The mobile robotic platform shown in Figure 1 is used to record the data of different sensors onboard. In particular, the FROG dataset provides the data of the front-mounted 2D LiDAR, along with annotations about the people in the field of view of the sensor (180 ° ), at a maximum distance of 10 m. We select this maximum distance due to the sparsity of LiDAR points (around 4 cm separation) making it challenging to reliably detect thin objects (people’s legs) beyond such distances. The odometry data is also provided.

**FIGURE 2:**
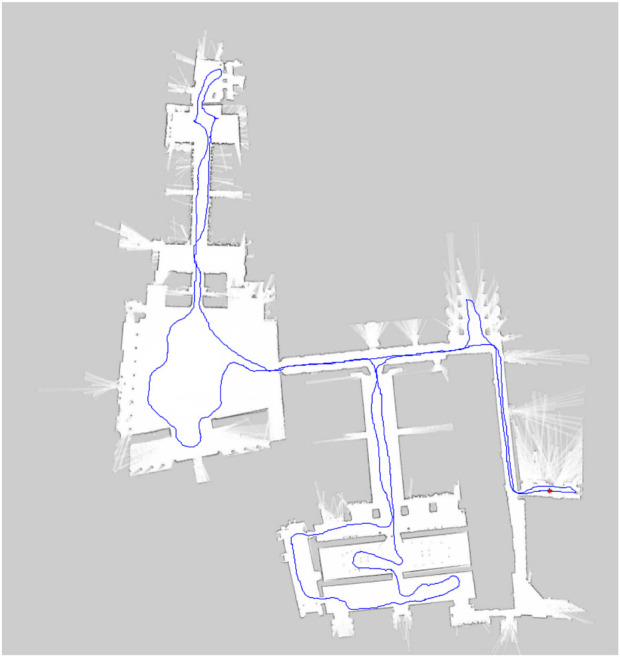
Example navigation plan used by the robot during capture of the FROG dataset.

The recorded data encompasses different time slots along 4 days of experiments. Each sequence consists of a tour around the Alcázar. The trajectory of the robot during one such tour can be seen in Figure 2. Table 1 shows a summary of several features of the recorded sequences. Around 40% of the scans are recorded outdoors, while around 60% are indoors.

**TABLE 1:** General overview of the annotated sequences in the FROG dataset. Each session lasted about half an hour, and we report the total number of scans and annotated people in each sequence.

Start time	Duration	Travel distance	Scans	People
10:31	26 m 42 s	1666.00 m	64238	127600
11:36	29 m 41 s	1845.66 m	71417	214707
12:43	31 m 43 s	1970.69 m	76088	258298
14:57	29 m 09 s	1824.06 m	70062	133197
15:53	25 m 16 s	1569.47 m	60758	133023
16:41	29 m 29 s	1843.57 m	70923	153658
Total	2 h 52 m 30 s	10719.45 m	413486	1020483

We focus on comparing our FROG dataset against the DROW dataset, the only currently available dataset that has been used to evaluate and compare 2D laser based people detectors. Table 2 shows a detailed quantitative comparison. Although the DROW dataset includes more hours of recordings, only a very small portion of the data is annotated–only 5.17% of the scans have associated annotations in the.wp files (this number includes empty lists of people). On the other side, the FROG dataset provides annotations for every single scan, a richer variety of crowded scenarios, over twice as many people per scan on average, and greatly increased laser/temporal resolutions. Moreover, our robot was able to move at faster navigation speeds than those achieved in DROW’s scenario, and thus traversed longer paths–over twice the distance compared to the odometry information provided by the DROW dataset.

**TABLE 2:** Comparison between the DROW and FROG datasets, showing different general metrics about each, and also including a comparison of the 2D LiDAR sensor used by the robots.

Scenario	DROW	FROG *(ours)*
	Elderly care facility	Royal Alcázar of Seville
Total scans	464013	413486
Annotated scans	24012	413486
Populated scans	14339	292889
Recorded time	ca. 10 h	ca. 3 h
Travel distance	5.18 km	10.72 km
People annotations	28984	1020483
Avg. # people/scan	1.2	2.5
Max. # people/scan	17	16
Laser model	SICK S300	Hokuyo UTM-30LX
Laser frequency	ca. 13 Hz	40 Hz
Laser height	37 cm	35 cm
Laser points	450	720
Laser FoV	225 °	180 °
Laser resolution	0.5 °	0.25 °

### 3.1 Laser scan labeling tool

Recording data from a robot typically involves using the ROS framework, which provides many facilities for interacting with device drivers, such as capturing data from the robot’s sensors. In ROS, the data of the 2D laser range finder sensors is provided through the structure given by the message sensor_msgs/LaserScan. In a nutshell, the range data is expressed as an array of distances in meters. Each position in the array can be mapped to a specific laser angle using metadata included in the ROS message, specifically the minimum/maximum angles covered by the sensor and the angle increment between measures. Finally, captured data from one or more sensors is usually stored in a format known as a *ROS bag file*.

We present our graphical tool used to annotate the dataset. It loads ROS bag files containing ROS laser scan messages, and graphically displays them using a top-down projection. The tool also allows visualizing image messages from a camera side by side (sensor_msgs/Image) if they are available either in the same ROS bag file or in an external time-synchronized bag file. This can help the user identify the people to be labeled in the scene. The scan labeler is implemented using Python 3, PyQt5 and ROS Noetic. It is publicly available on GitHub^3^, where a detailed description and instructions for use are included.

**FIGURE 3:**
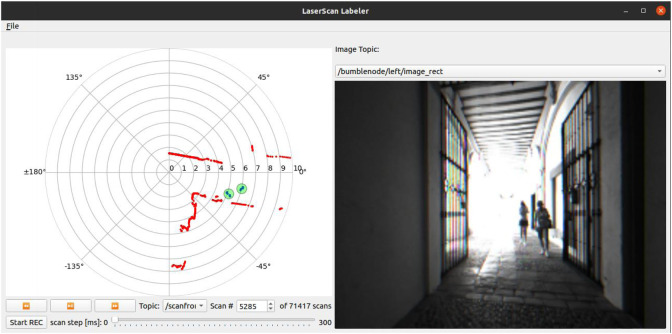
Main interface of the laser scan labeling tool. The tool displays the laser scan and the video feed from a camera topic side by side, and allows the user to easily create and track annotations using the mouse.

The main interface of the application can be seen in Figure 3. On the left side panel we can observe the projected laser scan (in red). The scan can then be labeled by creating/removing annotation circles enclosing the laser points that correspond to each person using the mouse. The user can at any time create, move, modify the radius or delete any circle. Moreover, the tool includes several options for playing back the laser scans and moving forwards/backwards in time at different speeds. Support for additional annotation classes is also present, like baby strollers, wheelchairs or other walking aids, and intended for future use.

An important feature of our tool is the ability to track the group of points inside each circle through time. When the user advances from one scan to the next, the center point of each circle is automatically recomputed as the mean of the points that still fall within the circle, which allows tracking each labeled person. This simple tracking does not depend on any automatic detection and is supervised by a human annotator to correct or restart it in case the tracking fails or non-person scan points are included in the circles. The tool does not enforce a minimum number of LiDAR points to create or track a circle–the human annotator is in charge of creating or deleting them appropriately based on the LiDAR information, as well as additional information such as the visual image or intuition. Our tool also assigns an internal identifier to each tracklet that the user creates, and these identifiers are temporally consistent for each tracklet (but there are no globally consistent IDs). Thanks to this feature, the annotation circles move along with people in subsequent scans, which considerably simplifies and speeds up the annotation process. The annotators are entirely responsible for resolving edge cases. For example, when people stand close by, the annotators were instructed to shrink the circles to contain only the scan points for each individual; avoiding grouping more than one person in the same circle and handling some partial occlusions.

In the specific case of the FROG dataset, the workload is distributed across four human annotators, all members of our laboratory group. Each annotator is in charge of annotating one or two ROS bag files. The work is carried out using our tool, annotating each bag in several sessions of around 10000 scans, taking breaks in between.

The output of the annotation tool is the list of circles associated with every annotated scan. Each circle includes the person tracklet identifier, center position (in Cartesian coordinates), and circle radius. Besides the list of circles, the timestamp and index of the scan within the sequence is also included so that the annotations can be traced to the original bag. In addition, the tool also supports generating segmentation data from the circles, containing the classification of each point in the laser scan data.

Finally, the tool supports exporting the aforementioned data in different file formats: .csv, .json, .mat (Matlab) and.npy (NumPy). This variety of formats is intended to make human review of the data easier, as well as subsequent loading in post-processing scripts. The finalized format of the FROG dataset is explained in the following section.

### 3.2 Format

The FROG dataset is finally delivered as a series of HDF5-formatted (The HDF Group, 2024) files. We use this file format in order to enable greater data loading efficiency, because HDF5 is specifically designed to store and organize large amounts of data, supporting partial/random access and easily integrating into Python NumPy code.

We make available a collection of Python scripts and modules in order to facilitate the loading process, as part of our benchmarking suite described in Section 5. This also includes the scripts we used to process the ROS bag files and the annotated scan data generated using our labeling tool, as well as exporting the data into the final HDF5 files; so that other researchers may be able to replicate our methodology with their own data.

We define the following arrays (known as *datasets* in HDF5 parlance):

• scans: This is a float32 array of dimension (N,720) containing each individual laser scan vector, where N is the total number of scans in the file. Each individual value is measured in meters.
• timestamps: This is a float64 array of dimension (N) containing the timestamp of each scan (in seconds since the UTC Unix epoch).
• circles: This is a float32 array of dimension (M,6) containing all person annotations, where M is the total number of person annotations in the file. The second dimension contains six values as following, specifying the position (in both cartesian and polar coordinates) of the person.
  • 0 and 1: Specifies the X/Y position (in meters) of the person.
  • 2: Specifies the radius (in meters) of the bounding circle that surrounds the person.
  • 3 and 4: Specifies the angle (in radians) and distance from the origin (in meters) of the person.
  • 5: Specifies the half-angle that covers the bounding circle when projected from the origin.

Person annotations are associated with only a single scan, and the exact range of entries in the circles array that correspond to each scan is defined by the circle_idx and circle_num arrays (explained below).

• circle_idx: This is an uint32 array of dimension (N) that specifies the index into circles of the first person annotation associated with each scan, the other annotations being stored sequentially afterwards.
• circle_num: This is an uint32 array of dimension (N) that specifies the total number of person annotations associated with each scan.
• split: This is an uint8 array of dimension (N) exclusive to the file containing the benchmark training/validation sets, specifying which scans belong to which set (0 = training, 1 = validation). The suggested split roughly follows a 90:10 proportion.

**FIGURE 4:**
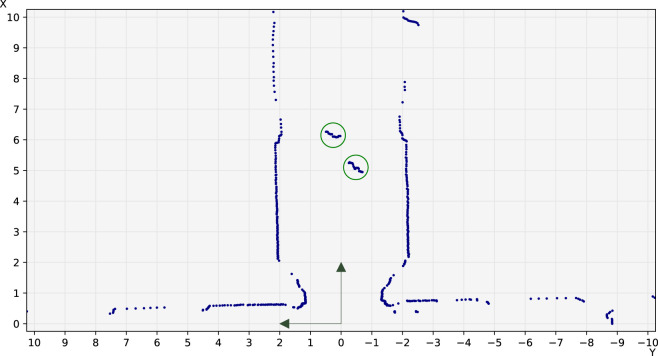
Example annotated laser scan showing the coordinate system used in the FROG dataset, matching the standard conventions used in robotics. The distances shown are in meters. Blue dots: points from the scan. Green circles: annotated people.

An important thing to note is that we follow the standard axis convention in robotics (see Figure 4). That is, the X-axis points forward, the Y-axis points left, and positive angles are counterclockwise. This causes the laser scan vectors to effectively be stored right-to-left.

Besides the HDF5-formatted files, we also make available the raw CSV files created with our labeling tool. These files contain partial people tracklets, meaning a single person may be associated with multiple tracklets depending on occlusions and other factors that affect the labeling process. Although existing 2D LiDAR people detection works (including this work) do not make use of them, we believe they may be useful to future researchers interested in the tracking approach.

Finally, we also make available the odometry data from each session as separate files in.npz (compressed NumPy) format. Each file contains two arrays, ts (containing timestamps) and data (containing X-position, Y-position and Z-rotation odometry samples for each timestamp). Like Beyer et al. (2017), the values are relative to an arbitrary initial state of the robot–only the differences between samples are meaningful. The odometry samples are not aligned with the scans due to differences in sample rate, and it is up to downstream users to devise a way to interpolate the state of the robot at each scan timestamp. Users should also keep in mind the relationship between the base frame of the robot, and the mounted laser frame, as explained in Section 3.

## 4 People detection

In addition to the FROG dataset, we propose a new end-to-end deep learning network that can detect people from 2D laser scan data. This network is inspired by image-based object detection networks such as Faster-RCNN (Ren et al., 2015) or the YOLO (Redmon et al., 2016) family of detectors, and motivated by the lack of approaches that are fully based on deep learning, instead relying on hand-crafted (non-deep) pre-processing and post-processing steps that need to be performed outside accelerators (GPU and TPU), such as the cutout generation and vote aggregation processes introduced by Beyer et al. (2017). We theorize those non-deep processes to be a source of processing speed bottlenecks (especially when performed on weak edge CPUs), which limits their ability to run on a robot’s built-in hardware. There are two contributions in our proposal: a network that can learn to extract features from 2D range finder data for use with downstream tasks, and a grid-based people detection head similar to RPN (Ren et al., 2015).

### 4.1 Laser Feature Extractor (LFE)

A 2D laser scan reading is usually presented as a 1D vector of range measurements, the position of each element within which determining the angle of the laser beam with respect to the origin. Deep learning algorithms learn to extract a set of abstract features about their input (as opposed to a specific set of features designed by humans), and use those features to solve a given problem (such as classification).

**FIGURE 5:**
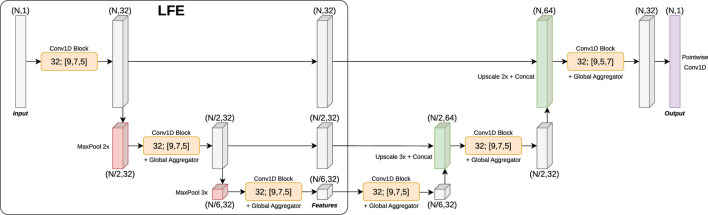
Laser Feature Extractor (LFE) network architecture, applied to a segmentation task. Each 1D convolutional block consists of three consecutive depthwise separable (Chollet, 2017) 1D convolutions of different kernel sizes (9, 7 and 5 respectively). Some blocks also contain a global feature aggregator, which performs a global maxpool of the input and concatenates the resulting features to each individual position of the input. Finally, a residual path adds the input of the block to the output of the last convolution. The segmentation mask is generated by an “inverse” LFE similar to U-Net (Ronneberger et al., 2015) followed by a pointwise convolution that produces the final output logits.

We propose a new Fully Convolutional Network, called the Laser Feature Extractor (LFE), which extracts features from a 1D vector of range measurements. This network is inspired by image classification and segmentation networks such as U-Net (Ronneberger et al., 2015), ResNets (He et al., 2016) or MobileNet (Howard et al., 2017). Its architecture (shown in Figure 5) consists of a stack of residual/convolutional and maxpool downscaling layers used to extract a feature map. There are three residual blocks in total, each containing three convolutional layers. All intermediate convolutional layers have 32 filters each. In order to reduce the search space and improve runtime speed, all convolutional layers are depthwise separable (Chollet, 2017): this means they are decomposed into two steps: a stack of independent convolutions (one applied to each corresponding channel), followed by a pointwise convolution. The activation function used after each convolution is ReLU, followed by batch normalization and dropout layers.

LFE generates features at three different levels of downsampling: the original resolution of the range data, the data downsampled by 2, and the data downsampled by 6 (in other words, combined downsampling by 2 and 3). This is especially useful given the polar nature of range data, thus allowing features to be extracted at close distances (where the input resolution is bigger) and also at farther distances (where the input resolution is smaller). The downsampling factors have been chosen to increase the likelihood of evenly dividing the number of points in the laser scan vector (for instance, DROW’s 450 points cannot be divided by 4).

#### 4.1.1 Training protocol

While the backbone of an object detector is traditionally pretrained with a simpler classification problem (and dataset such as ImageNet (Deng et al., 2009)), there is no such equivalent available for 2D laser scan data. In order to validate LFE on its own, we consider the laser scan segmentation problem (similar to PeTra (Guerrero-Higueras et al., 2019)), and use it to allow LFE to learn relevant features for detecting people. In order to use LFE in a segmentation problem, we attach an “inverse LFE”, making the whole network similar to U-Net (Ronneberger et al., 2015). This network thus learns a binary label for each input point, identifying which points are part of people’s legs (and which are not). The segmentation output can also be post-processed with classical algorithms (such as SciPy’s find_peaks function) in order to generate discrete people detections, something which we will revisit in a later section of this paper.

### 4.2 People Proposal Network (PPN)

**FIGURE 6:**
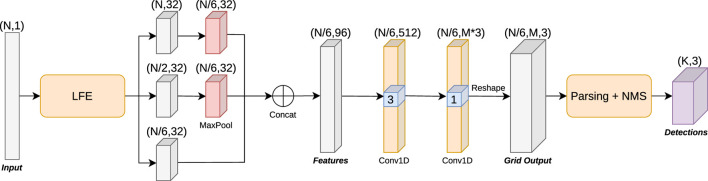
People Proposal Network (PPN) architecture, incorporating a LFE backbone. The features extracted by the LFE are further processed by a depthwise separable (Chollet, 2017) 1D convolutional layer with kernel size 3, after which the outputs ( M×3 ) for each sector in the grid are generated by a final pointwise 1D convolutional layer. The Non Maximum Suppression (NMS) process parses the grid output and generates the final people detections.

**FIGURE 7:**
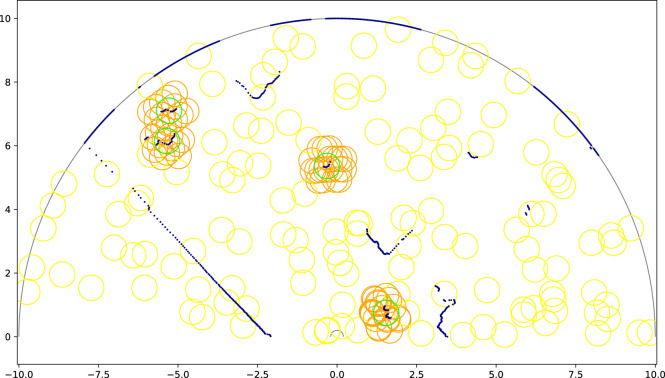
Example anchor grid used by the People Proposal Network during training on the FROG dataset. The boundaries of the field of view are drawn in gray. The laser scan data is represented by navy blue dots. Green circles are ground truth people annotations. Orange and yellow circles are examples of positive and negative anchor circles respectively (not all shown). An anchor is considered as positive ( s=1 ) if it is close enough to a ground truth person.

The second component of the network is the People Proposal Network (PPN). This network (shown in Figure 6) is directly inspired and based on the Region Proposal Network (RPN) introduced by the foundational object detection work Faster-RCNN (Ren et al., 2015). We adapt the anchor grid of object proposals of the RPN so that it can be used in the fundamentally 1D problem of laser scan data people detection. In the original RPN the anchor grid is bidimensional; each element of the feature map represents a 2D subarea of the original image, and several anchors are trained in parallel for each 2D subarea using different aspect ratio priors. In our People Proposal Network the anchor grid corresponds to sectors of the full field of view of the laser, with their amplitude and number determined by the largest downsampling performed by LFE. We place multiple anchors at each sector, each having different distance priors fully and evenly covering the entire range of the depth axis. The field of view of the PPN has the shape of a circular ring sector (see Figure 7). An important consequence of this design is that anchors are more densely placed in central (near) areas of the field, while being sparser at far areas. This is due to dealing with a polar coordinate system as opposed to a Cartesian coordinate system–this is in line with the nature of 1D laser scan data.

In mathematical terms, the center point of each anchor in the grid corresponds to the polar coordinate $\left(\theta_{i},r_{j}\right)$ . Given N (number of angular sectors), M (number of depth sectors), φ (angular field of view of the laser), $r_{\text{min}}$ (minimum depth) and $r_{\text{max}}$ (maximum depth); we define $\theta_{i}=-\frac{1}{2}\varphi +\left(\frac{1}{2}+i\right)\frac{\varphi }{N}$ with i=0,1,…,N−1 , and $r_{j}=r_{\text{min}}+\left(\frac{1}{2}+j\right)\frac{r_{\text{max}}-r_{\text{min}}}{M}$ with j=0,1,…,M−1 . Afterwards, we can define the Cartesian coordinates $P_{ij}=\left(r_{j}\cos\theta_{i},r_{j}\sin\theta_{i}\right)$ .

The PPN receives the feature maps extracted by LFE as input, and outputs three target values for each anchor: s (objectness), Δd (distance offset) and Δℓ (arc offset). The feature maps generated at different downsampling levels are maxpooled into the same resolution and concatenated in order to generate a single input feature map. The objectness score is learned as a classification problem, while the distance/arc offsets are learned as a regression problem. These offsets are centered on each individual anchor’s center point. We learn the arc offset instead of the angle offset so that the learned offsets are in the same displacement scale as the distance offsets, instead of having drastically different scales depending on how close to the origin each individual anchor is. Furthermore, we normalize their scale by dividing both offsets by the anchor spacing along the depth axis: $\frac{d_{\text{far}}-d_{\text{near}}}{N_{\text{anchors}}}$ , where $d_{\text{near}}$ and $d_{\text{far}}$ are the minimum and maximum detection distances considered, respectively. We also empirically show that resulting target offsets used for learning have a close-to-normal distribution ( $\mu_{\Delta d},\mu_{\Delta \ell }\approx 0,\sigma_{\Delta d},\sigma_{\Delta \ell }\approx 1$ ), see Table 3.

**TABLE 3:** Statistical information (mean and standard deviation) about the two regression targets in the generated training data: distance offset ( Δd ) and arc offset ( Δℓ ).

Target	μ	σ
Δd	0.0605	0.9166
Δℓ	0.0019	0.8773

At inference time, the distance and arc offsets of each anchor are decoded into Cartesian XY coordinates representing the center of a bounding circle, and paired with their corresponding classification scores. Note that these centers are usually located between the two legs of a person, and they do not necessarily correspond to individual sensor measurements; in fact they rarely do (if ever). As in Beyer et al. (2017); Jia et al. (2020), all circles are defined to have the same radius. Like Ren et al. (2015), the output from the network then undergoes a Non-Maximum Suppression (NMS) filter. Traditional NMS as applied in object detection is based on the Intersection over Union (IoU) measurement between bounding box proposals. In our case we use a simple distance function between person center proposals. In other words, two person proposals overlap if the distance between their centers is smaller than a given hyperparameter, which usually matches the most common ground truth circle diameter.

#### 4.2.1 Training protocol

The training process of the network involves generating anchor classification and regression data for every scan, based on the person annotations in the ground truth. In a similar way to Ren et al. (2015), we group all anchors in the grid into two categories: positive and negative. The overlap metric used as criterion is once again the distance between circle centers, and the boundary between groups is a tunable hyperparameter.

The loss function used is the following:

$$L=\frac{1}{N_{+}+N_{-}}L_{\mathrm{cls}}+\frac{1}{N_{+}}L_{\mathrm{reg}}$$

where $L_{\mathrm{cls}}$ is the classification loss, $L_{\mathrm{reg}}$ is the Smooth L1 regression loss and $N_{+}$ / $N_{-}$ are the number of positive/negative anchors respectively. Positive and negative anchors both contribute to classification loss, while only positive anchors contribute to regression loss. Both losses are scaled so that they have the same magnitude, by virtue of dividing by the number of contributing anchors respectively. Given the large imbalance between positive and negative anchors, we combine the traditional binary cross-entropy loss with the Dice loss (Sudre et al., 2017) (a smooth variant of the $F_{1}$ score), taking the average of the two (Galdran et al., 2023) as classification loss. We do not follow the approach in Ren et al. (2015) (resampling the positive and negative anchors within each scan to be in a fixed 1:1 proportion, with each scan producing the same overall number of anchors) because said imbalance is greater than the one in 2D object detection, causing that approach to be rendered unfeasible. This is also an effect of the unevenness of anchor density with respect to distance, meaning a large number of scans do not contain enough positive anchors to properly fill the desired quota.

## 5 FROG benchmark

We propose using the FROG dataset as a new benchmark for 2D laser range finder based people detectors. As such, we carry out several experiments with existing detectors, as well as our own proposed detectors. In particular, we select the DROW3 (Beyer et al., 2018), DR-SPAAM (Jia et al., 2020) and PeTra (Guerrero-Higueras et al., 2019) detectors from the state of the art for an initial benchmark based on the FROG dataset, in addition to a well known baseline provided by the ROS framework (Pantofaru, 2010). The benchmark codebase we developed to perform these experiments can be found on GitHub^4^, and it provides a common implementation of all metrics and evaluation protocols for maximum consistency.

We define a subset of the FROG dataset to be used in this benchmark, containing training/validation and testing sets. The training/validation set is sourced from two different sequences recorded around the time of greatest attendance (around noon, maximizing the number of person annotations) and later randomly split in 90:10 proportion. The testing set is sourced from another different sequence. In both cases, scans with empty lists of person annotations are excluded from the benchmark. Models are trained on the training set, and metrics are calculated and reported on the testing set. The validation set is only used to provide feedback during the training process, as well as optimizing hyperparameters. We provide all the data, and do not withhold the labels associated with the testing set.

### 5.1 Evaluation criteria and process

We follow existing practices in Beyer et al. (2017), Beyer et al. (2018), Jia et al. (2020), Jia et al. (2021), and use the same metrics for evaluation purposes. These metrics revolve around the Precision-Recall (PR) curve, which is intended to show the overall performance profile of the model at different desired precision/recall tradeoffs. Particularly, we consider the following:

• Average Precision: This is the main evaluation metric used by the object detection community, and it is nominally equivalent to the area under the PR curve (AuC). However, estimating this area can be a challenging process due to discontinuities created by small variations in example ranking. For this reason, we follow the object detection community (specifically MS COCO (Lin et al., 2014)) in using the 101-recall-point interpolation method to calculate this metric. This contrasts with Beyer et al. (2017), which applied the trapezoidal rule instead. As a note, we believe this metric produces unexpected behavior when evaluating certain methods. In Section 5.3 this is explained with more detail.
• Peak F1 score: This is the maximum F1 score obtained along the PR curve. Note that the F1 score is the harmonic mean between the Precision and Recall values.
• Equal Error Rate (EER): This is the closest value along the PR curve at which Precision equals Recall.

These metrics can be parametrized: for example, AP_d_ considers detections to be positive if there exists an unmatched ground truth annotation within d m of the detection. This d parameter is known as the *association distance*, and it is analogous to the IoU threshold of object detection metrics. Note that only people centers are considered, as opposed to full circles. Following established 2D LiDAR people detection work (Beyer et al., 2018; Jia et al., 2020), we calculate the PR curve and evaluate all associated metrics using two different values for d : 0.5 m and 0.3 m. In addition, we calculate averaged mAP, mPeak F1 and mEER metrics for d=[0.3:0.05:0.5] m in order to capture overall performance at different association distances with a single value, similarly to MS COCO and its mAP metric over a range of IoU thresholds.

In order to calculate the PR curve, we first obtain the collection of person detections produced by each model for each scan in the tested split. Each person detection is expected to have a confidence score (0.0–1.0), and X/Y positions. We ignore very low confidence detections (with score < 0.01), as well as detections falling outside a 10 m distance threshold; so that the evaluation process takes a reasonable amount of time, and also to avoid unfairly rewarding methods that generate hundreds of noisy detections with very low confidence scores. Within each scan, detections are matched to their closest ground truth annotation. If multiple detections are matched to the same ground truth, the one closest to the annotation becomes a True Positive and the others are considered False Positives. Detections that are farther than d to any ground truth annotation are also considered False Positives. Following existing aforementioned works, the matchups between detections and ground truth circles are recalculated after processing every detection (in descending order of confidence score), so that higher confidence detections can initially count as True Positives in case a lower confidence detection (processed later) is closer to a ground truth annotation–this ultimately only replaces one detection for another, and does not change the total tally of True Positives. A final global aggregation process traverses the complete list of detections across scans (also in descending order), accumulating a count of True Positives and thus computing every (P,R) point of the curve. If multiple detections share the same confidence score, they are summarized as a single point in the PR curve.

Lastly, we also report the end-to-end inference time as an evaluation metric. Specifically, we use the ROS environment with each detector’s provided ROS node, and measure the total time taken by the node to process each laser scan and output detected people. This is calculated by playing back the test set at a laser frequency high enough to bottleneck all detectors, and dividing the simulated time by the number of received people detection messages. Concretely, we play back the test set at 20 times the speed, resulting in a 800 Hz laser frequency sustained for 88.49s.

### 5.2 Experimental setup

As mentioned previously, we select a baseline and several state-of-the-art detectors (as well as our own detectors) to be evaluated as part of the first benchmark using the FROG dataset. In this section we will discuss the exact methodology used to test each detector, including hyperparameters used, challenges encountered during evaluation, adaptations needed to produce meaningful results, and other miscellaneous details.

The platform used to train and evaluate models is a desktop PC sporting an Intel Core i9-9900X CPU with 128 GB of RAM and an NVIDIA TITAN RTX GPU with 24 GB of VRAM.

#### 5.2.1 ROS leg_detector baseline

ROS 1 distributions offer a standard package containing a pre-trained 2D laser based leg detector/person tracker. This detector implements a variant of Arras et al. (2007), a classical algorithm based on hand-crafted geometric segment features followed by a random forest classifier. We include its results in the benchmark because it is a commonly used solution in the field of robotics, making it relevant as a baseline to which compare the performance of other detectors. In order to more accurately represent the baseline as typically employed in robotics projects, we follow Beyer et al. (2018) and use the provided pre-trained forest as-is (with all default hyperparameters); i.e., not retraining the model with data from the FROG dataset. People tracking measurement results are captured via ROS bag, and later converted into the common evaluation format expected by our benchmarking codebase.

#### 5.2.2 PeTra

We evaluate PeTra (Guerrero-Higueras et al., 2019) as a modern representative of leg-based detectors. This work essentially replaces ROS leg_detector’s classical algorithm with an image-based 2D fully convolutional segmentation network, which detects points belonging to people’s legs. This segmentation output is later post-processed in order to extract individual leg locations, and pairs detected legs to produce final person location proposals. The source code was made available online by its authors^5^.

We train PeTra’s segmentation network using 512×256 as the resolution of the 2D image onto which the laser scan data is projected, with the height dimension being half of the width dimension in order to save computational resources (given that the FoV of the laser is 180° , and otherwise the bottom half of the image would always remain unused). In addition, we randomly sample a set of 12000 scans from FROG’s training dataset to convert into projected 2D images instead of converting them all, due to the increased memory and computation requirements of working with 2D images as opposed to 1D laser scan vectors.

We train the network for 30 epochs with a batch size of 16 scans using the Adam optimizer with $\eta =5\times 10^{-4}$ .

**FIGURE 8:**
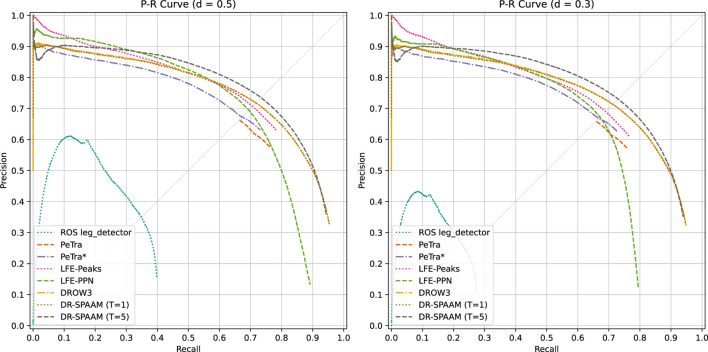
Precision-recall curves for the person detector models. We show curves for both association distances: 0.5 m and 0.3 m.

PeTra uses OpenCV’s findContours routine to find shapes corresponding to legs; however it does not generate a detection score. In order to solve this, we take the average output of the network associated with each point belonging to each contour as its overall detection score. Unfortunately, due to the fact that PeTra is trained using Dice loss, many points end up with the maximal 1.0 score allowed by the output of the sigmoid (after the inevitable loss of precision), meaning that a large number of detections are generated with confidence 1.0. This results in a very short PR curve, as can be seen in Figure 8. To remedy this problem, we also evaluate PeTra with the same mixed loss function used by our own segmentation-based detector, resulting in a model we called PeTra*. This allows us to lower the overconfidence of the detections, and thus plot a more meaningful PR curve for PeTra.

We perform inference speed tests using PeTra’s provided Docker image based on ROS 1 Melodic. We do not evaluate PeTra* separately because it only differs in model weights. In order to ensure accurate results, we set the subscriber queue length to 1, and move the detector code into the subscriber callback (the original code performs detections as a separate timer task, independent of laser frequency).

#### 5.2.3 DROW3 & DR-SPAAM

We evaluate DROW3 (Beyer et al., 2018) and DR-SPAAM (Jia et al., 2020) as representatives of the cutout-based input preprocessing approach. In particular, we evaluate the most up to date implementation of both models^6^ with minimal modifications to the code in order to add support for loading the FROG dataset. These modifications include adding custom training configuration files and disabling the code that deletes certain log folders–this allows us to directly read the generated detections and run our common benchmarking code, which is shared with all other models we have evaluated.

We train and evaluate both DROW3 and DR-SPAAM in single-scan mode, that is, no information from previous scans is used to generate predictions. We do this in order to enable a fair comparison with other models that do not make use of temporal information. In addition, we also train and present results for DR-SPAAM using a temporal window size of 5 scans, so that said approach is also represented in the benchmark. DROW3 cannot be evaluated in multi-scan mode because the codebase used for training does not support reading odometry information. We use the same cutout hyperparameters selected in Jia et al. (2020) for optimal person detection, in particular: 56 points (1.0 m × 1.0 m), window size.

We train the networks for 5 epochs with a batch size of 8, the Adam optimizer, and with an exponential learning rate schedule ranging from $10^{-3}$ initially to $10^{-6}$ at the end of training. In particular, we use 5 epochs so that the overall number of minibatches processed during training on the FROG dataset is of the same order of magnitude as when training using the DROW dataset.

We perform inference speed tests using ROS 1 Noetic, the provided dr_spaam_ros package, and the latest available version of PyTorch at the time of writing (v2.0.1 with CUDA support). “DR-SPAAM*” is not included in the comparison due to not being included in the ROS package provided by the authors.

#### 5.2.4 LFE & PPN

We first train LFE on the segmentation problem described in Section 4.1.1, using a batch size of 32 and the AdamW (Loshchilov and Hutter, 2019) optimizer with $\eta =10^{-3}$ and $\lambda =4\times 10^{-3}$ . We set a target of 100 epochs, with an early stopping patience of 20 epochs and $\Delta L_{\min}=10^{-3}$ . As mentioned previously, we also evaluate LFE on its own as a person detector by adding a classical post-processing step based around SciPy’s find_peaks function, a combination we are calling LFE-Peaks. The post-processing finds the height and width of the peaks in the segmentation signal, and afterwards computes centroids based on the Cartesian coordinates of the corresponding points in the range data. Centroids that are close together are interpreted as legs or part of legs, and merged together into final person detections using a NMS-like process. Outlier points are discarded from each centroid, so that we do not take into account parts of the background in the averaging formula. In a similar way to our adaptation of PeTra, the confidence score is the average output of the network corresponding to all points assigned to each detection.

LFE-PPN embeds LFE as its backbone, which can either be trained from scratch, or its weights reused from the LFE segmentation experiment and further fine-tuned during LFE-PPN training. For the purposes of this experiment, we follow and report the latter approach, although we have not observed any quantitative differences between the two. We employ a batch size of 4, use the AdamW optimizer with $\eta =10^{-4}$ and $\lambda =4\times 10^{-4}$ , and set a target of 150 epochs with an early stopping patience of 20 epochs and $\Delta L_{\min}=10^{-3}$ .

We perform inference speed tests using ROS 2 Humble and our own developed implementation of a person detection node based on either LFE-Peaks or LFE-PPN, using C++ and ONNX Runtime v1.14.1^7^ with the CUDA backend. This runtime framework is selected because of its intended use as a standalone inference engine with direct compatibility with C++, and its ability to use multiple backends tailored to different ML accelerators, such as those available on edge devices. In the case of LFE-Peaks, an embedded Python interpreter is used to execute the classical peak finding and post processing algorithm. Our ROS package is publicly available on GitHub^8^.

### 5.3 Results

We present the quantitative results of the benchmark (shown in Table 4), as well as the corresponding Precision-Recall curves in Figure 8. Methods based on cutout preprocessing (DROW3 and DR-SPAAM) obtain the overall best metrics, as expected of the state of the art; while our own proposed methods (LFE and PPN) achieve good results considering how they utilize fewer or no hand-crafted features in their design. The ROS leg_detector baseline produces very poor results, clearly indicating the end of its usefulness after the development of much better detectors; however they are consistent with the results obtained by Beyer et al. (2018) on the DROW dataset.

**TABLE 4:** Benchmark results for several person detector models. Besides the baseline, all models are trained on FROG’s train split, and evaluated on FROG’s test split. Average Precision (AP), Peak $F_{1}$ and Equal Error Rate (EER) are reported for two association distances: 0.5 m and 0.3 m, as well as averaged across [0.3:0.05:0.5]m . End-to-end inference times obtained with each detector’s ROS node are also reported, in milliseconds.

Method				d = 0.5 m			d = 0.3 m			Time (ms)
	mAP	mPeak $F_{1}$	mEER	AP	Peak $F_{1}$	EER	AP	Peak $F_{1}$	EER	
ROS leg_detector	15.8	30.8	30.5	20.2	35.2	34.9	10.0	24.3	24.1	1.77
PeTra	49.6	66.4	66.2	50.1	66.6	66.4	49.1	66.1	65.9	28.17
PeTra*	58.3	67.7	67.1	59.0	67.9	67.3	57.9	67.4	66.8	—
LFE-Peaks (ours)	64.9	70.2	70.2	65.6	70.7	70.7	63.2	69.0	69.0	1.76
LFE-PPN (ours)	66.5	68.7	68.6	69.2	69.5	69.5	62.5	67.3	67.2	1.49
DROW3 (T=1)	73.6	71.9	71.6	73.9	72.0	71.8	73.0	71.6	71.4	13.08
DR-SPAAM (T=1)	73.3	71.9	71.6	73.7	72.1	71.8	72.7	71.6	71.3	13.95
DR-SPAAM (T=5)	75.3	73.4	73.3	75.6	73.6	73.4	74.7	73.2	73.0	13.99

Counter-intuitive behavior can be observed in the behavior of the Average Precision metric. We suspect this metric rewards methods with a longer PR curve, said length being a result of incorporating a larger number of low confidence/quality guesses, which serendipitously inflates the maximum recall score (at the expense of precision). Methods which do not generate such guesses are unfairly punished. Another reason for this effect has to do with the nature of score outputs. Networks trained using binary crossentropy loss do not reach the extreme values (1.0, 0.0), and thus they produce many more data points for the Precision-Recall curve, while networks trained with other loss functions (such as PeTra’s Dice loss) may saturate towards the extremes due to loss of precision caused by extreme logit value outputs. For this reason we believe the Peak-F1 and EER metrics present a fairer comparison in practice.

We can observe that the PR curves and metrics of DROW3 and DR-SPAAM in single scan mode are practically identical. We believe this to be related to the lack of temporal information used during training, indicating that the contributions in Jia et al. (2020) are not focused on improving the baseline network architecture introduced by Beyer et al. (2018). On the other hand, full DR-SPAAM with a temporal window size of 5 scans achieves the best metrics overall, although its PR curve dips below that of DROW3 in the low recall section.

PeTra presents a challenging problem during evaluation. As explained earlier, its choice of loss function generates a large number of detections with the maximum possible confidence score (1.0), causing a significant portion of the data to be lumped together as a single point, which in turn results in a degenerate PR curve. We correct this by changing the loss function to incorporate binary crossentropy. The resulting model (PeTra*) does not present this problem, and thus achieves results more in line with the ones obtained by other models. Despite being limited in the size of the training set, its results are fairly reasonable.

LFE-Peaks and LFE-PPN manage to outperform the state of the art in the low recall/high precision zone of the PR graph. LFE-Peaks in particular is able to trade blows against LFE-PPN and be competitive on its own against DROW3, however it loses in mAP and AP @ 0.5 m due to the length of its PR curve (stopping at less than 80% recall, while LFE-PPN is able to cross that threshold). These results clearly indicate that the 1D convolutional layers in LFE are capable of competently learning people-identifying features directly from range data. The PPN shows results that clearly validate the idea of replacing classical post-processing algorithms with a fully deep approach inspired by object detection, however it still requires further tuning and improvements to be able to outperform the state of the art. Of note is its difference in performance depending on the associating distance, which we theorize could be caused by shortcomings in the design of the regression targets, or the distribution of the training data; thus indicating the need to further adjust people center proposal generation.

In terms of inference speed, we can observe large differences between processing times achieved by each detector. Some implementations (leg_detector and our LFE-Peaks/LFE-PPN nodes) take less than 2 ms to process each scan. Comparing these three detectors, there is a difference of about 0.3 ms between LFE-PPN and the other two–in other words, LFE-PPN is 15% faster. DR-SPAAM takes around 14 ms (similar to the time reported in Jia et al. (2020)) regardless of temporal window size thanks to its auto-regressive architecture. DROW3’s network architecture is slightly faster than DR-SPAAM’s, taking around 13 ms. Finally, PeTra takes around 28 ms. Some of these large differences could be attributed to a variety of reasons not necessarily related to model architecture, such as differences in the software technologies used. For instance, DROW3/DR-SPAAM’s ROS node is fully implemented in Python and PyTorch (which in turn includes rospy serialization/deserialization overhead), while the rest are all implemented in C++. PeTra uses TensorFlow v1.x–a long since deprecated branch that is no longer maintained, while our LFE/PPN nodes use ONNX Runtime–a library specifically designed for fast inference times in deployed applications. In any case, this comparison involves real systems that are currently available for use by robotics researchers.

**FIGURE 9:**
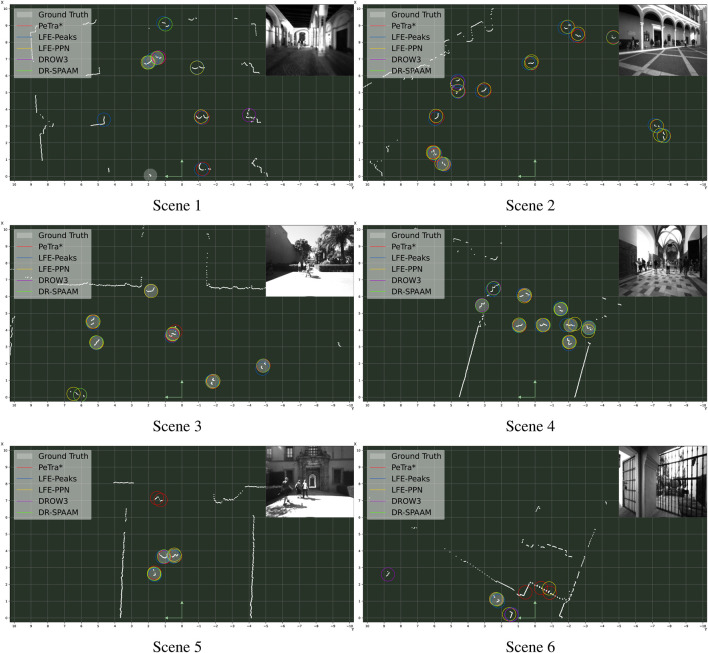
Collection of qualitative results, sampled from the FROG dataset’s test set. In the case of PeTra and DR-SPAAM, we only show the best variant (PeTra* and DR-SPAAM with T = 5) for clarity. Several scenes showcasing the performance of the detectors in different types of environments are included.

Finally, we qualitatively evaluate the detectors. We provide a video^9^ showing the laser scan sequence (with the reference image feed from the robot in the upper right corner), and plotted circles corresponding to both the ground truth and each detector. We only plot detections whose confidence is greater or equal than a given threshold matching the confidence of the point at the Peak-F1 score of each detector. Stills from the video can be seen in Figure 9, showing several types of environments: indoors (Scenes 1, 4, 6), outdoors (2, 3, 5), crowded (4), with challenging geometry (1, 2, 6). The most interesting thing to note about the detectors is their different ways of producing false positives. Certain kinds of challenging geometry (such as pillars, fences or wall corners) can cause models to incorrectly predict the presence of people. Models that do not aggregate information from several scans tend to make more mistakes in these situations.

## 6 Conclusions and future work

We showcased a brand new dataset for people detection using 2D range finders called FROG, as well as the process and tools we used to semi-automatically carry out the annotation process. We also proposed our own deep learning based people detectors leveraging this data; and afterwards we designed, implemented and carried out a benchmark intended to evaluate people detectors using the FROG dataset. We obtained and reported results for a collection of state-of-the-art detectors, and commented on the performance of each one. We can draw certain conclusions: 2D LiDAR-based person detection is still an open problem, and we hope we contribute to it through our dataset and our proposed models.

As future work, we intend to improve our models so that a fully deep learning based approach (without non-deep pre-processing and classical post-processing steps) can surpass the performance of existing models, besides already providing a faster implementation. Moreover, we plan to focus our efforts on the speed and usability of the models when executed directly on a real robot platform with low power on-device AI accelerators, as opposed to a separate desktop system with powerful hardware. Regarding the dataset, we recorded more sequences than we annotated. This opens the possibility of extending the dataset in the future, and using the new data as a hidden test set for competition purposes. Moreover, the FROG dataset is well suited to supporting further analysis of the generalization capability of people detectors, as the nature of its scenario generates difficult targets, such as far away or fast moving people. Finally, we propose further exploring the potential of self-supervised approaches (Jia et al., 2021; Arreghini et al., 2025), as well as fusing detection results from different sensor sources for a combined integral approach to people detection.

## Data Availability

The datasets presented in this study can be found in online repositories. The names of the repository/repositories and accession number(s) can be found below: https://robotics.upo.es/datasets/frog/laser2d_people/.

## Footnotes

1
https://robotics.upo.es/datasets/frog/laser2d_people/

2
https://robotics.upo.es/datasets/frog/upo/

3
https://github.com/robotics-upo/laserscan_labeler

4
https://github.com/robotics-upo/2DLaserPeopleBenchmark

5
https://github.com/ClaudiaAlvarezAparicio/petra

6
https://github.com/VisualComputingInstitute/2D_lidar_person_detection

7
https://onnxruntime.ai/

8
https://github.com/robotics-upo/upo_laser_people_detector

9
https://youtu.be/czzddmL1pLI

## References

[B1] Arras K. O. Mozos Ó. M. Burgard W. (2007). Using boosted features for the detection of people in 2D range data in IEEE International Conference on Robotics and Automation (ICRA), 3402–3407.

[B2] Arreghini S. Carlotti N. Nava M. Paolillo A. Giusti A. (2025). Sixth sense: indoor human spatial awareness dataset. 10.5281/ZENODO.14936068

[B3] Beyer L. Hermans A. Leibe B. (2017). DROW: real-time deep learning based wheelchair detection in 2D range data. IEEE Robotics Automation Lett. (RA-L) 2, 585–592. 10.1109/lra.2016.2645131

[B4] Beyer L. Hermans A. Linder T. Arras K. O. Leibe B. (2018). Deep person detection in two-dimensional range data. IEEE Robotics Automation Lett. (RA-L) 3, 2726–2733. 10.1109/lra.2018.2835510

[B5] Caesar H. Bankiti V. Lang A. H. Vora S. Liong V. E. Xu Q. (2020). “nuScenes: a multimodal dataset for autonomous driving,” in IEEE Conference on Computer Vision and Pattern Recognition (CVPR).

[B6] Chavdarova T. Baqué P. Bouquet S. Maksai A. Jose C. Bagautdinov T. (2018). “WILDTRACK: a multi-camera HD dataset for dense unscripted pedestrian detection,” in IEEE Conference on Computer Vision and Pattern Recognition (CVPR), 5030–5039. 10.1109/CVPR.2018.00528

[B7] Chollet F. (2017). “Xception: deep learning with depthwise separable convolutions,” in IEEE Conference on Computer Vision and Pattern Recognition (CVPR), 1251–1258.

[B8] Cong P. Zhu X. Qiao F. Ren Y. Peng X. Hou Y. (2022). “STCrowd: a multimodal dataset for pedestrian perception in crowded scenes,” in IEEE Conference on Computer Vision and Pattern Recognition (CVPR), (Los Alamitos, CA, USA), 19576–19585. 10.1109/CVPR52688.2022.01899

[B9] Deng J. Dong W. Socher R. Li L.-J. Li K. Fei-Fei L. (2009). “ImageNet: a large-scale hierarchical image database,” in IEEE Conference on Computer Vision and Pattern Recognition (CVPR), 248–255. 10.1109/CVPR.2009.5206848

[B10] Evers V. Menezes N. Merino L. Gavrila D. Nabais F. Pantic M. (2014). “The development and real-world deployment of FROG, the fun robotic outdoor guide,” in ACM/IEEE International Conference on Human-Robot Interaction (ACM), 100. 10.1145/2559636.2559649

[B12] Galdran A. Carneiro G. Ballester M. A. G. (2023). “On the optimal combination of cross-entropy and soft dice losses for lesion segmentation with out-of-distribution robustness,” in Diabetic foot ulcers grand challenge (Cham), 40–51.

[B13] Geiger A. Lenz P. Stiller C. Urtasun R. (2013). Vision meets robotics: the KITTI dataset. Int. J. Robotics Res. (IJRR) 32, 1231–1237. 10.1177/0278364913491297

[B14] Guerrero-Higueras A. M. Álvarez Aparicio C. Calvo Olivera M. C. Rodríguez-Lera F. J. Fernández-Llamas C. Rico F. M. (2019). Tracking people in a Mobile robot from 2D LIDAR scans using full convolutional neural networks for security in cluttered environments. Front. Neurorobotics 12, 85. 10.3389/fnbot.2018.00085 PMC6332292 30670960

[B15] He K. Zhang X. Ren S. Sun J. (2016). “Deep residual learning for image recognition,” in IEEE Conference on Computer Vision and Pattern Recognition (CVPR). CVPR ’16, 770–778. 10.1109/CVPR.2016.90

[B16] Howard A. G. Zhu M. Chen B. Kalenichenko D. Wang W. Weyand T. (2017). MobileNets: efficient convolutional neural networks for Mobile vision applications. arXiv Prepr. arXiv:1704.04861.

[B17] Jia D. Hermans A. Leibe B. (2020). “DR-SPAAM: a spatial-attention and auto-regressive model for person detection in 2D range data,” in International Conference on Intelligent Robots and Systems (IROS), 10270–10277. 10.1109/iros45743.2020.9341689

[B18] Jia D. Steinweg M. Hermans A. Leibe B. (2021). “Self-supervised person detection in 2D range data using a calibrated camera,” in International Conference on Robotics and Automation (ICRA), 13301–13307. 10.1109/icra48506.2021.9561699

[B19] Karnan H. Nair A. Xiao X. Warnell G. Pirk S. Toshev A. (2022). Socially CompliAnt navigation dataset (SCAND): a large-scale dataset of demonstrations for social navigation. IEEE Robotics Automation Lett. (RA-L) 7, 11807–11814. 10.1109/LRA.2022.3184025

[B20] Kim W. Ramanagopal M. S. Barto C. Yu M.-Y. Rosaen K. Goumas N. (2019). PedX: benchmark dataset for metric 3-D pose estimation of pedestrians in complex urban intersections. IEEE Robotics Automation Lett. (RA-L) 4, 1940–1947. 10.1109/LRA.2019.2896705

[B21] Lin T.-Y. Maire M. Belongie S. Hays J. Perona P. Ramanan D. (2014). “Microsoft COCO: common objects in context,” in European Conference on Computer Vision (ECCV) (Cham), 740–755. 10.1007/978-3-319-10602-1_48

[B22] Loshchilov I. Hutter F. (2019). “Decoupled weight decay regularization,” in International Conference on Learning Representations (ICLR).

[B23] Martín-Martín R. Patel M. Rezatofighi H. Shenoi A. Gwak J. Frankel E. (2023). JRDB: a dataset and benchmark of egocentric robot visual perception of humans in built environments. IEEE Trans. Pattern Analysis Mach. Intell. 45, 6748–6765. 10.1109/tpami.2021.3070543 33798067

[B24] Pantofaru C. (2010). ROS leg detector package. Available online at: https://wiki.ros.org/leg_detector.

[B25] Ramón-Vigo R. Pérez-Lara J. Caballero F. Merino L. (2014). “Navigating among people in crowded environment: datasets for localization and human robot interaction Workshop on robots in clutter: perception and interaction in clutter,” in IEEE/RSJ International Conference on Intelligent Robots and Systems (IROS).

[B26] Redmon J. Divvala S. Girshick R. Farhadi A. (2016). “You only look once: unified, real-time object detection,” in IEEE Conference on Computer Vision and Pattern Recognition (CVPR), 779–788. 10.1109/CVPR.2016.91

[B27] Ren S. He K. Girshick R. Sun J. (2015). “Faster R-CNN: towards real-time object detection with region proposal networks,” in Advances in neural information processing systems, 28. 10.1109/TPAMI.2016.2577031 27295650

[B28] Ronneberger O. Fischer P. Brox T. (2015). “U-Net: Convolutional networks for biomedical image segmentation,” in Medical image computing and computer-assisted intervention (MICCAI) (Cham), 234–241.

[B29] Rudenko A. Kucner T. P. Swaminathan C. S. Chadalavada R. T. Arras K. O. Lilienthal A. J. (2020). THÖR: human-robot navigation data collection and accurate motion trajectories dataset. IEEE Robotics Automation Lett. (RA-L) 5, 676–682. 10.1109/lra.2020.2965416

[B30] Schreiter T. Almeida T. Zhu Y. Gutiérrez Maestro E. Morillo-Mendez L. Rudenko A. (2022). “The magni human motion dataset: Accurate, complex, multi-modal, natural, semantically-rich and contextualized,” in IEEE International Conference on Robot & Human Interactive Communication (United States). 10.48550/arXiv.2208.14925

[B31] Sudre C. H. Li W. Vercauteren T. Ourselin S. Jorge Cardoso M. (2017). “Generalised dice overlap as a deep learning loss function for highly unbalanced segmentations,” in Deep learning in medical image analysis and multimodal learning for clinical decision support (Cham), 240–248. 10.1007/978-3-319-67558-9_28 PMC7610921 34104926

[B34] The HDF Group (2024). “Hierarchical Data Format, version 5”. https://github.com/HDFGroup/hdf5.

[B32] Xie Z. Dames P. (2024). Semantic2D: a semantic dataset for 2D lidar semantic segmentation. Dataset. 10.5281/ZENODO.13730199

[B33] Yang H. Yang Y. Yao C. Liu C. Chen Q. (2024). Li2Former: omni-dimension aggregation transformer for person detection in 2-D range data. IEEE Trans. Instrum. Meas. 73, 1–12. 10.1109/TIM.2024.3420353

